# Characterization of the transition to oral feeding in premature newborns

**DOI:** 10.1590/2317-1782/20212021136

**Published:** 2022-04-20

**Authors:** Jaianne Lourdes Furtado Costa, Ana Paula Sabino de Medeiros Neves, Juliana Dantas de Araújo Santos Camargo, Raquel Coube de Carvalho Yamamoto

**Affiliations:** 1 Programa de Residência Multiprofissional em Terapia Intensiva Neonatal, Universidade Federal do Rio Grande do Norte – UFRN - Natal (RN), Brasil.; 2 Unidade de Cuidados Intermediários Canguru, Maternidade Escola Januário Cicco – MEJC - Natal (RN), Brasil.; 3 Setor de Regulação e Avaliação em Saúde, Maternidade Escola Januário Cicco – MEJC - Natal (RN), Brasil.; 4 Departamento de Fonoaudiologia, Universidade Federal do Rio Grande do Norte – UFRN - Natal (RN), Brasil.

**Keywords:** Premature Infant, Feeding Behavior, Breast Feeding, Feeding Methods, Enteral Nutrition

## Abstract

**Purpose:**

To characterize the transition from alternative feeding methods to oral feeding, to investigate techniques to favor feeding used during the transition, and the prevalence of breastfeeding at hospital discharge of premature newborns.

**Methods:**

The following variables were considered: gender, gestational age and birth weight, classification of prematurity, time of transition to oral use, corrected gestational age and newborn weight at the beginning and end of the food transition, transition technique used, length of hospitalization and type of breastfeeding at discharge. For data analysis, the software SPSS version 25.0 was used considering the significance level of 5%.

**Results:**

Significant results were observed between the premature groups for corrected gestational age at the beginning of the food transition, transition time, and hospital days.

**Conclusion:**

The study concludes that the breast-probe technique was the most used. The length of hospital stay was shorter for the group of late and moderate preterm newborns. The transition time to the oral route was longer in the group of very preterm infants. Moreover, the proportion of exclusive breastfeeding at hospital discharge was similar between the prematurity classification groups.

## INTRODUCTION

Oral feeding can be a challenging process for preterm newborns (PTNB)^(1)^ that present immaturity of the oral-motor sensory system and, consequently, lack of coordination between suction, deglutition, and breathing functions during feeding. Associated with this, clinical instability of breathing patterns, prolonged intubation periods, and immaturity of the gastrointestinal system, lead to the use of alternative feeding methods^(2-4)^, which can interfere with breastfeeding^(2,5)^ due to difficulties related to adequate grip on the breast and extraction of milk while feeding^(6)^.

For oral feeding to occur safely and effectively, the newborn must present primitive reflexes (searching, GAG, bite, and suction), which allow for the localization of food and the protection of airways during deglutition^(7)^. For this, some characteristics such as tongue cupping, adequate jaw movement, blocks, force and rhythm of suction, signs of awareness of the infant and coordination between suction, deglutition, and breathing, as well as factors such as weight, clinical stability, corrected gestational age, organ maturity, and behavioral state should be taken into account when beginning oral feeding to avoid the risk of aspiration and to allow for hospital discharge^(6,8)^.

The use of stimulation techniques for nutritive suction, which assist in the maturation of the oral-motor-sensory system, favor the safe transition to oral feeding (TOF) and reduce the transition period, achieving exclusively oral feeding quicker^(9-12)^. TOF is understood to be the process that starts when the infant, currently using alternative feeding methods, begins oral feeding (focusing on breastfeeding) and achieves exclusively oral feeding^(8,11)^.

According to the World Health Organization, during the first six months of life, an infant should exclusively breastfeed, continuing up to two or more years. Breastfeeding provides diverse benefits for both mother and infant including physical, emotional, neurological, and cognitive aspects^(13)^, in addition to assisting in the development of the stomatognathic system^(2,14)^. Therefore, it is believed that the younger the gestational age at birth, the longer the transition to oral feeding, possibly reducing the chances of exclusive breastfeeding at discharge. Given this, this research aimed to characterize the transition from alternative to oral feeding, investigating the techniques that favor feeding and the prevalence of breastfeeding at the discharge of premature newborns.

## METHODS

The present research is an observational, longitudinal, and prospective study developed in the Neonatal Intensive Care Unit (NICU) and at the Kangaroo Intermediate Care Unit (KICU) of the Januário Cicco Maternity School, with the implementation of the Friends of the Children Hospital Initiative (FCHI). The research was approved by the Research Ethics Committee, under process number 3.311.874, according to the recommendations of resolution 466/2012 of the National Health Council. Responsible guardians agreed to participate in the research and signed a Free and Informed Consent Form.

The inclusion criteria for the sample were: premature newborns with gestational age at birth of less than 37 weeks, being cared for at the NICU and/or KICU by the speech therapy team during the period from January to June 2020, who had undertaken the transition from alternative feeding to oral breastfeeding. Patients with a diagnosis of III- or IV-degree peri-intraventricular hemorrhage, neurological alterations, heart disease, craniofacial deformities, bronchopulmonary dysplasia, anoxia rated with APGAR below 7 in the fifth minute, and syndromes were excluded.

The data collected were: gender, gestational age and weight at birth, corrected gestational age and weight at the start and finish of TOF, total time (in days) necessary for feeding transition, as well as the technique used during transition. Additionally, information regarding hospitalization time and discharge were recorded, along with information for weight, corrected gestational age, and form of feeding at the time of discharge.

The sample was stratified into three groups according to the prematurity classification^(15)^, with the first group including late PTNBs (gestational age between 34 and 36 weeks and 6 days); the second with moderate PTNBs (gestational age between 32 and 33 weeks and 6 days); and the third group consisting of very premature newborns (gestational age between 28 and 31 weeks and 6 days).

For PTNBs to be able to safely start oral feeding, the criteria for clinical stability and weight, starting from 1300g, were adopted in the NICU and KICU where the research was carried out. In these two units, after satisfying these two criteria, the speech therapist evaluated if the NB was ready to start TOF, intervening in the transition process, as well as suggesting the removal of the alternative feeding tube. During the transition process from feeding with the enteral tube to oral feeding, the speech therapist undertook therapeutic work aiming at exclusive breastfeeding where possible, in addition to managing the feeding transition in collaboration with the multidisciplinary team.

For the statistical analysis, the Shapiro-Wilk normality test was applied seeking to verify agreement of the continuous variables with the normal distribution. The descriptive analysis of the variables was realized with the averages and standard deviation (Average ± SD), median, 25^th^ and 75^th^ percentiles, and absolute and relative frequencies. To evaluate the difference for the continuous variables and that those presented normality between the prematurity groups, one-way ANOVA and Tukey-Kramer post hoc tests were used. For the variables that did not present normality, the Kruskal-Wallis test was performed and, subsequently, comparisons of pairs were realized using the Dunn procedure (1964) with Bonferroni correction for multiple comparisons. The Chi-square test was used to analyze the association between the categorical variables. A significance level of 5% was adopted for all analyses. The SPSS software (Statistical Package for the Social Sciences, Chicago, EUA) version 25.0, was used for the analyses.

## RESULTS

Information was collected from the hospital records of 52 premature infants who satisfied the inclusion criteria, with 32 female (61.53%) and 20 male (38.46%) babies.

A difference between the medians for corrected gestational age at the start of the TOF for the prematurity groups, (p < 0.01) (Table 1) was observed. The post hoc analysis showed significant differences in the medians between the late and very PTNB groups (p < 0.01) and between the moderate and late PTNB groups (p = 0.002), but not between the moderate and very PTNB groups (p = 0.142).

**Table 1:** Characterization of the transition from alternative feeding to oral feeding by prematurity classification

**Variables**	**Prematurity**			p-value^a^
	**Late**	**Moderate**	**Very pre-term**	
	**(N=18)**	**(N=16)**	**(N=18)**	
Birth Data				
GA (weeks)	35 (34 – 35)	33 (32 – 33)	30 (29 – 31)	< 0.01*
Weight (grams)	1.729 (± 233)	1.741 (± 372)	1.431 (± 256)	0.003*
Start of TOF				
CGA (weeks)	36 (35 – 36)	34 (33 – 35)	33 (32 – 34)	< 0.01*
Weight (grams)	1.638	1.735	1.550	0.052
	(1.558 – 1.775)	(1.648 – 1.895)	(1.454 – 1.696)	
End of TOF				
CGA (weeks)	37 (36 – 38)	35 (35 – 36)	35 (34 – 36)	< 0.01*
Weight (grams)	1.793	1.841	1.848	0.588
	(1.712 – 1.937)	(1.797 – 1.968)	(1.721 – 2.027)	
		
Total time of TOF (days)	7 (6 – 11)	10 (7 – 18)	12 (11 – 17)	0.009*
				

a Significance of difference between the groups by ANOVA or Kruskal-Wallis tests

The continuous data are expressed in average and standard deviation or median and 25^th^ and 75^th^ percentiles

* Significance at p < 0.05

Abbreviations: GA, gestational age; TOF, transition to oral feeding; CGA, corrected gestational age

There was a difference for corrected gestational age medians at the end of the TOF for the stratified groups (p < 0.01) (Table 1). The post hoc analysis showed significant differences for the medians between the late and very PTNB groups (35) (p < 0.01), and between the moderate and late PTNB groups (p = 0.006), but not between the moderate and very PTNB groups (p = 0.490).

Concerning the duration of the TOF, a difference between the medians of the groups was observed (p = 0.009) (Table 1). The post hoc analysis showed significant differences between the medians for the late and very PTNB groups (p = 0.007), but not between the late and moderate PTNB groups (p = 0.306) nor between the moderate and very PTNB groups (p = 0.542).

Of the 52 infants, 67.3% went to the NICU before going to the KICU. There was a difference between the medians of the groups for hospitalization time in the NICU (p < 0.01) (Table 2). The post hoc analysis showed significant differences in the medians between the late and very PTNB groups (p < 0.01) and between the moderate and very PTNB groups (p = 0.001), but not between the moderate and late PTNB groups (p = 1.000).

**Table 2:** Hospitalization time by prematurity classification

**Variables**	**Prematurity**			p-value^a^
	**Late**	**Moderate**	**Very pre-term**	
	**(N=18)**	**(N=16)**	**(N=18)**	
**NICU (%)**				
Yes	9 (50.0)	8 (50.0)	18 (100.0)	**0.001** *
No	9 (50.0)	8 (50.0)	0 (0.0)	
**Hospitalization time (days)**				
NICU	1 (0 – 10)	3 (0 – 10)	16 (12 – 25)	p < 0.01*
KICU	15 ± 7	16 ± 6	26 ± 9	0.002*
NICU + KICU	20 (15 – 24)	23 (17 – 31)	43 (34 – 48)	p < 0.01*

a Significance of difference between the groups by ANOVA or Kruskal-Wallis tests (continuous variables) or Pearson Chi-square test (categorical variables)

The continuous data are expressed in averages and standard deviation or median and 25^th^ and 75^th^ percentiles

* Significance in p < 0.05

Abbreviations: NICU, Neonatal Intensive Care Unit; KICU, Kangaroo Intermediate Care Unit

The length of stay in the KICU was statistically different between the prematurity groups (p = 0.002). The post hoc Tukey-Kramer analysis showed that the difference in time in the KICU between the very and late PTNB groups was statistically significant (p = 0.002), as well as the difference between the moderate and very PTNB groups (p = 0.011).However, there was no significant difference between the time in the KICU for the moderate and late PTNB groups (p = 0.914).

The results for the technique used during the transition from alternative to exclusively oral feeding method and behavior on discharge are presented by prematurity classification (Table 3).

**Table 3:** Technique used for feeding transition and feeding behavior at discharge by prematurity classification

**Variables**	**Prematurity**			p-value^a^
	**Late (N=18)**	**Moderate (N=16)**	**Very pre-term (N=18)**	
Technique (%)				
Translactation	2 (11.1)	3 (18.8)	4 (22.2)	0.667
Breast-probe	16 (88.9)	13 (81.2)	14 (77.8)	
**Behavior at discharge** (%)				
EB	14 (77.8)	12 (75.0)	14 (77.8)	0.976
MB + COMPL	4 (22.2)	4 (25.0)	4 (22.2)	

a Significance of difference between the groups by Pearson Chi-square test

Abbreviations: EB, Exclusive breastfeeding; MB, maternal breast; COMPL, complement

## DISCUSSION

The weight and corrected gestational age at which to start TOF are still widely discussed. In some neonatal units, these aspects represent criteria to begin oral feeding for premature newborns^(8,12,16)^. In the present study, it was found that the median age to start TOF for the sample varied from 32 to 36 weeks for corrected gestational age. Despite not being criteria in the maternity ward where the research was undertaken, corrected gestational age is an important indicator for the maturity of the oral function of NBs in terms of coordination between suction, deglutition, and respiration functions, which are essential for safe oral feeding^(2,8)^. However, it does not need to be understood as a determining factor to initiate this process^(17)^.

Regarding gestational age at birth, the results of the present study suggest that the more immature the newborn, that is, the lower the gestational age at birth, the longer the TOF. This situation could be related to the organic immaturity of the PTNB, which leads to reduced coordination between suction, deglutition, and respiration and, given this, the need for alternative feeding methods to guarantee nutrition^(3)^. Despite the long transition time to oral feeding for very premature newborns, the prevalence of exclusive breastfeeding at discharge was similar between the groups. This shows that the recommendation to maintain maternal milk extraction as well as the technique used by the speech therapist to favor oral feeding could have been factors that contributed to favoring maternal breastfeeding at discharge, independent of the gestational age at birth of the infant.

Of the techniques used, the feeding breast-probe technique was most frequently applied. This technique consists in feeding exclusively by gastric tube/gavage feeding (breast-probe), without the use of a cup or bottle^(11)^. When this stimulation takes place safely, that is, when the infant presents an adequate level of suction and clinical stability, the TOF occurs as early as possible through suction, reducing the time for tube feeding and leading to exclusively oral feeding^(17)^. Frequently, PTNBs are not ready to extract the necessary amount of milk they need for nutrition. However, with the training of suction on the maternal breast, the newborn will improve their ability to coordinate suction, deglutition, and breathing and favor the tonicity and mobility of the articulatory phonetics organs^(11,12)^. In clinical practice, the breast-probe feeding technique has shown itself beneficial for providing suction without external interference, which can generate the “nipple confusion” for grip such as when using a bottle and/or cup. This favors continual and gradual learning of natural suction on the maternal breast, which favors adequate craniofacial development of the child, stimulates and maintains exclusive maternal breastfeeding, and promotes proximity between the mother-infant pair.

The translactation technique^(17,18)^, used in 17.3% of PTNB in the sample, seeks to make the adaptation of the infant to the breast more physiological, favoring milk production and establishing coordination between suction, deglutition, and breathing. This technique is used in TOF of PTNB allowing for the association of milk ingestion received from the tube with the suction of the infant during feeding^(17,19)^, thereby stimulating learning of suction to favor breastfeeding in this population.

Providing feeding by cup, bottle, or finger-feeding technique during TOF was not observed in this research. Due to being a Friends of the Children Hospital Initiative (FCHI), the practice of breastfeeding was encouraged by the whole team. Given that the offer of milk by the cup is not the most physiological option, principally when used over the long term, we agree that suction should be stimulated like suction on the breast^(9)^. For this, the translactation technique, as well as the enteral tube, are the options in which the PTNB will be trained and will receive milk through suction directly on the breast^(19)^.

Exclusive breastfeeding was the behavior that predominated at discharge. Three studies, with samples similar to this study, show that the prevalence of breastfeeding for premature infants at discharge was 85.2%^(20)^, 58.3%^(21)^, and 23.3%^(22)^. The three studies were realized in hospitals with FCHI. However, in comparing the studies, divergent values for the prevalence of exclusive breastfeeding in infants were observed. It is worth noting that the strategies considered by the whole team of professionals involved favor breastfeeding. Additionally, special needs for patients with some type of physical impairment, or craniofacial or neurological anomaly, are factors that should be considered in the difficulties for achieving breastfeeding at discharge. One of these studies presented important results, showing that even with an 85.7% prevalence of PTNB with exclusive breastfeeding at discharge, 15 days after discharge, prevalence dropped to 75%, and 30 days after discharge continued to drop, reaching 46.3%^(20)^. Highlighting the importance of including strategies to monitor breastfeeding management even after discharge, allows both the mother and infant to benefit from exclusive breastfeeding in the first 6 months of corrected age.

The length of stay in the NICU and KICU was greater for the very premature group. This prolonged stay in the NICU, owing to the diverse neonatal complications resultant from immaturity, can generate an impact on neuro-psychomotor development and is considered an important factor in developmental delays due to reduced adequate sensory input^(23)^. Therefore, the prolonged stay in the NICU could be one of the causes for the longer TOF in the very premature preterm group, in addition to being related to the higher rates of mixed maternal breastfeeding^(19)^. Positively, in the maternity ward where the research was realized, the speech therapist is the professional who evaluates if oral feeding can be implemented safely for the PTNB. The input of this professional in the multidisciplinary team assists in the TOF process so that the transition is safe and effective, seeking to achieve exclusive maternal breastfeeding on discharge.

The literature regarding TOF in premature infants, techniques used and prevalence of breastfeeding in this population is scarce. It is important that future studies are realized that investigate the limitations during this process and the difficulties encountered to achieve exclusive breastfeeding at discharge.

## CONCLUSION

This study found that the Breast-probe feeding technique was the most commonly utilized during the transition from enteral tube feeding to oral feeding and that the transition time was quicker for the late and moderate preterm newborns, responding positively to the hypothesis that the younger the gestational age at birth, the longer the transition time to oral feeding.

It was also possible to observe that the majority of premature newborns were discharged exclusively breastfeeding, responding negatively to the hypothesis since it was found that the probability of observing exclusive breastfeeding at discharge was similar between the 3 prematurity classification groups.

## References

[1] Brasil (2017). Atenção humanizada ao recém-nascido: Método Canguru: manual técnico.

[2] Alves YVT, Santos JCJ, Barreto IDC, Fujinaga CI, Medeiros AMC (2019). Avaliação da sucção não nutritiva de recém-nascidos a termo e sua relação com o desempenho da mamada. Rev Bras Saúde Mater Infant.

[3] Amoris EVN, Nascimento EN (2020). Transição alimentar em prematuros: fatores interferentes. Rev CEFAC.

[4] Pagliaro CL, Bühler KEB, Ibidi SM, Limongi SCO (2016). Dietary transition difficulties in preterm infants: critical literature review. J Pediatr.

[5] Lubbe W (2018). Clinicians guide for cue‐based transition to oral feeding in preterm infants: an easy‐to‐use clinical guide. J Eval Clin Pract.

[6] Levy DS, Almeida ST, Castelli CTR, Levandowisk DC, Almeida ST (2018). Aleitamento materno em situações de risco para disfagia..

[7] Fujinaga CI, Maltauro S, Stadler ST, Cheffer ER, Aguiar S, Amorin NEZ (2018). Estado comportamental e o desempenho da prontidão do prematuro para início da alimentação oral. Rev CEFAC.

[8] Prade LS, Bolzan GP, Berwig LC, Yamamoto RCC, Vargas CL, Silva AMT (2016). Relação entre prontidão para início da alimentação oral e desempenho alimentar em recém-nascidos pré-termo. Audiol Commun Res.

[9] Moreira CM, Cavalcante-Silva RP, Fujinaga CI, Marson F (2017). Comparison of the finger-feeding versus cup feeding methods in the transition from gastric to oral feeding in preterm infants. J Pediatr (Rio J).

[10] Arora K, Goel S, Manerkar S, Konde N, Panchal H, Hegde D (2018). Prefeeding oromotor stimulation program for improving oromotor function in preterm infants – A Randomized Controlled Trial. Indian Pediatr.

[11] Medeiros AMC, Ramos BKB, Bomfim DLSS, Alvelos CL, Silva TC, Barreto IDC (2018). Tempo de transição alimentar na técnica sonda-peito em recém-nascidos baixo peso do Método Canguru. CoDAS.

[12] Cavalcante SEA, Oliveira SIM, Silva RKC, Sousa CPC, Lima JVH, Souza NL (2018). Habilidades de recém-nascidos prematuros para início da alimentação oral. Rev Rene..

[13] Brasil (2015). Saúde da Criança: aleitamento materno e alimentação complementar.

[14] Cassimiro IGV, Souza PG, Rodrigues MC, Carneiro GKM (2019). A importância da amamentação natural para o sistema estomatognático. Rev UNINGÁ.

[15] Sociedade Brasileira de Pediatria (2017). Prevenção da prematuridade: uma intervenção da gestão e da assistência.

[16] EBSERH (2018). Procedimento Operacional Padrão: avaliação da prontidão para transição de dieta para via oral em recém-nascido pré-termo (RNPT).

[17] Rego JD (2015). Aleitamento materno..

[18] Brasil (2018). Método canguru: diretrizes do cuidado.

[19] Pessoa-Santana MCC, Silveira BL, Santos ICS, Mascarenhas MLVC, Dias EGC (2013). Métodos alternativos de alimentação do recém-nascido: considerações e relato de experiência. Rev Bras Ciênc Saúde..

[20] Lima APE, Castral TC, Leal LP, Javorski M, Sette GCS, Scochi CGS (2019). Aleitamento materno exclusivo de prematuros e motivos para sua interrupção no primeiro mês pós-alta hospitalar. Rev Gaúcha Enferm.

[21] Czechowski AE, Fujinaga CI (2010). Seguimento ambulatorial de um grupo de prematuros e a prevalência do aleitamento na alta hospitalar e ao sexto mês de vida: contribuições da Fonoaudiologia. Rev Soc Bras Fonoaudiol.

[22] Arns-Neumann C, Ferreira TK, Cat MNL, Martins M (2020). Aleitamento materno em prematuros: prevalência e fatores associados à interrupção precoce. Jornal Paranaense de Pediatria..

[23] Alemdar DK, Inal S (2020). The effect of individualized developmental care practices in preterm infant. Complement Med Res.

