# Supplementary material for: Characterization of the transition to oral feeding in premature newborns
Source: Codas. 2022 Apr 20;34(5):e20210136. doi: 10.1590/2317-1782/20212021136 (PMC9886178; doi:10.1590/2317-1782/20212021136)
Supplement: Translation

# Translation: Caracterização da transição alimentar para via oral em recém-nascidos prematuros

**Authors:** Jaianne Lourdes Furtado Costa, Ana Paula Sabino de Medeiros Neves, Juliana Dantas de Araújo Santos Camargo, Raquel Coube de Carvalho Yamamoto

**Affiliations:** 1 Programa de Residência Multiprofissional em Terapia Intensiva Neonatal, Universidade Federal do Rio Grande do Norte – UFRN - Natal (RN), Brasil.; 2 Unidade de Cuidados Intermediários Canguru, Maternidade Escola Januário Cicco – MEJC - Natal (RN), Brasil.; 3 Setor de Regulação e Avaliação em Saúde, Maternidade Escola Januário Cicco – MEJC - Natal (RN), Brasil.; 4 Departamento de Fonoaudiologia, Universidade Federal do Rio Grande do Norte – UFRN - Natal (RN), Brasil.

**Keywords:** Recém-nascido Prematuro, Comportamento Alimentar, Aleitamento Materno, Métodos de Alimentação, Nutrição Enteral

## Abstract

**Objetivo:**

Caracterizar a transição alimentar da via alternativa para via oral, investigar as técnicas para favorecer a alimentação e a prevalência de aleitamento materno na alta hospitalar de recém-nascidos prematuros.

**Método:**

Estudo observacional, longitudinal e prospectivo desenvolvido numa maternidade. Amostra foi composta por 52 recém-nascidos prematuros e estratificada entre os grupos de prematuridade tardio, moderado e muito pré-termo. Consideraram-se as variáveis: gênero, idade gestacional e peso de nascimento, classificação da prematuridade, tempo de transição para via oral, idade gestacional corrigida e peso do recém-nascido no início e final da transição alimentar, técnica de transição utilizada, tempo de internação hospitalar e tipo de aleitamento no momento da alta. A análise estatística foi realizada com o Software SPSS, versão 25.0. Considerou-se o nível de significância de 5%.

**Resultados:**

Foram observados resultados significativos entre os grupos com relação à idade gestacional corrigida no início da transição alimentar, tempo de transição alimentar da via alternativa para via oral e dias de internação.

**Conclusão:**

O estudo conclui que a técnica sonda-peito foi a mais utilizada. O tempo de internação foi menor para o grupo de recém-nascidos pré-termo tardio e moderado. O tempo de transição alimentar para via oral maior no grupo de prematuros muito pré-termo e a proporção do aleitamento materno exclusivo na alta hospitalar foi semelhante entre os grupos de classificação da prematuridade.

## INTRODUÇÃO

A alimentação por via oral é um processo que pode ser complexo para o recém-nascido pré-termo (RNPT)^(1)^, que pode apresentar imaturidade do sistema sensório motor-oral e, consequentemente, incoordenação entre as funções de sucção, deglutição e respiração no momento da alimentação. Associado a isso, a instabilidade clínica do padrão respiratório, tempo prolongado de intubação e imaturidade do sistema gastrointestinal, levam ao uso de uma via alternativa de alimentação^(2-4)^, podendo interferir no aleitamento^(2,5)^ devido às dificuldades para uma pega adequada no seio materno e a extração de leite na mamada^(6)^.

Para que ocorra uma alimentação por via oral de forma segura e eficaz é necessário que o recém-nascido apresente reflexos primitivos (procura, GAG, mordida e sucção), que permitem localizar o alimento e protegem as vias aéreas durante a deglutição^(7)^. Para isso, algumas características são necessárias como o canolamento de língua, movimentos adequados de mandíbula, blocos, força e ritmo de sucção, sinais de alerta do bebê e coordenação entre a sucção, deglutição e respiração^(3,7)^. Além destes, fatores como peso, estabilidade clínica, idade gestacional corrigida, maturação de órgãos e sistemas e estado comportamental devem ser levados em consideração para iniciar a alimentação por via oral evitando o risco de aspiração e possibilitando a alta hospitalar^(6,8)^.

O uso de técnicas de estimulação de sucção nutritiva, que auxiliam a maturação do sistema sensório-motor-oral, favorecem a transição para via oral (TVO) de forma segura e reduzem o período de transição chegando à alimentação plena por via oral em menor tempo^(9-12)^. Entende-se por TVO o processo que tem início quando o bebê, em uso de via alternativa de alimentação, inicia a alimentação por via oral (com enfoque no aleitamento materno) e finaliza com a via oral plena^(8,11)^.

Segundo a Organização Mundial da Saúde, a amamentação deve ser exclusiva nos primeiros seis meses de vida do bebê e continuada até os dois anos ou mais, pois o aleitamento materno garante diversos benefícios à mãe e ao bebê envolvendo aspectos físicos, emocionais, neurológicos e cognitivos^(13)^, além de auxiliar no desenvolvimento do sistema estomatognático^(2,14)^. Sendo assim, acredita-se que quanto menor a idade gestacional de nascimento, maior o tempo de transição para via oral, podendo diminuir as chances de aleitamento materno exclusivo na alta hospitalar. Dessa forma, o objetivo da pesquisa foi caracterizar a transição alimentar da via alternativa para via oral, investigar as técnicas para favorecer a alimentação e a prevalência de aleitamento materno na alta hospitalar de recém-nascidos prematuros.

## MÉTODO

Trata-se de um estudo observacional, longitudinal e prospectivo desenvolvido na Unidade de Terapia Intensiva Neonatal (UTIN) e na Unidade de Cuidados Intermediários Canguru (UCINCA) da Maternidade Escola Januário Cicco, com implantação da Iniciativa Hospital Amigo da Criança (IHAC). A pesquisa teve aprovação pelo Comitê de Ética e Pesquisa, sob o parecer número 3.311.874, obedecendo às recomendações da resolução 466/2012 do Conselho Nacional de Saúde. Os responsáveis concordaram em participar da pesquisa e assinaram o Termo de Consentimento Livre e Esclarecido.

Os critérios de inclusão da amostra foram: recém-nascido prematuro com idade gestacional de nascimento inferior a 37 semanas, ser atendido na UTIN e/ou UCINCA no período de Janeiro a Junho de 2020 pela equipe de fonoaudiologia e ter realizado a transição alimentar da via alternativa para via oral em seio materno. Foram excluídos pacientes com diagnóstico de: hemorragia peri-intraventricular grau III e IV, alterações neurológicas, cardiopatia, deformidades craniofaciais, displasia broncopulmonar, anóxia classificada com APGAR abaixo de 7 no quinto minuto, e síndromes.

Os dados coletados foram: gênero, idade gestacional e peso de nascimento, idade gestacional corrigida e peso de início e final da TVO, tempo total (em dias) necessário para a transição alimentar, assim como a técnica utilizada durante a transição. Além disso, foram registradas informações sobre o tempo de internação e alta, sendo coletadas informações sobre o peso, idade gestacional corrigida e a forma de alimentação no momento da alta.

A amostra foi estratificada em três grupos conforme a classificação da prematuridade^(15)^, sendo o primeiro grupo com os RNPT tardios (idade gestacional entre 34 semanas e 36 semanas e 6 dias); o segundo com os RNPT moderados (idade gestacional entre 32 semanas e 33 semanas e 6 dias); e o terceiro composto pelos recém-nascidos muito pré-termo (idade gestacional entre 28 semanas e 31 semanas e 6 dias).

Para que o RNPT inicie a alimentação por via oral de forma segura, adotaram-se na UTIN e UCINCA da maternidade onde a pesquisa foi realizada os critérios de estabilidade clínica e peso, a partir de 1300g. Nessas duas unidades, após alcançar esses dois critérios, o fonoaudiólogo avalia se o RN está apto para iniciar a TVO, intervém no processo de transição, bem como sugere a retirada da via alternativa de alimentação. Durante o processo de transição da alimentação da sonda enteral para a alimentação por via oral, o fonoaudiólogo realiza o trabalho terapêutico objetivando o aleitamento materno exclusivo sempre que possível, além de fazer o gerenciamento da transição alimentar em concordância com a equipe multiprofissional.

Para a análise estatística, o teste de normalidade de Shapiro-Wilk foi aplicado visando verificar a aderência das variáveis contínuas à distribuição normal. A análise descritiva das variáveis foi realizada pela média e desvio-padrão (Média ± DP), mediana, percentis 25 e 75, frequências absolutas e relativas. Para avaliar a diferença das variáveis contínuas e que apresentaram normalidade entre os grupos de prematuridade, utilizou-se a Anova de um fator e teste post hoc de Tukey-Kramer. Para as variáveis que não apresentaram normalidade, o teste de Kruskal-Wallis foi realizado e, posteriormente, comparações de pares foram realizadas usando o procedimento de Dunn (1964) com uma correção de Bonferroni para comparações múltiplas. O teste Qui-quadrado foi utilizado para analisar a associação entre as variáveis de natureza categórica. O nível de significância de 5% foi adotado para todas as análises. O software utilizado para as análises foi o SPSS (Statistical Package for the Social Sciences, Chicago, EUA), versão 25.0.

## RESULTADOS

Foram coletadas informações do prontuário de 52 bebês prematuros que atenderam aos critérios de inclusão da pesquisa, sendo: 32 bebês do sexo feminino (61,53%) e 20 do sexo masculino (38,46%).

Foi evidenciada uma diferença entre as medianas da idade gestacional corrigida no início da TVO dos grupos de prematuridade, (p < 0,01) (Tabela 1). A análise post hoc mostrou diferenças significativas nas medianas entre os grupos de RNPT tardio e recém-nascido muito pré-termo (p < 0,01) e entre os grupos de RNPT moderado e tardio (p = 0,002), mas não entre os grupos de RNPT moderado e muito pré-termo (p = 0,142).

**Tabela 1:** Caracterização da transição da alimentação alternativa para via oral por classificação da prematuridade

**Variáveis**	**Prematuridade**			p-valor^a^
	**Tardio**	**Moderado**	**Muito pré-termo**	
	**(N=18)**	**(N=16)**	**(N=18)**	
Dados do Nascimento				
IG (semanas)	35 (34 – 35)	33 (32 – 33)	30 (29 – 31)	< 0,01*
Peso (gramas)	1.729 (± 233)	1.741 (± 372)	1.431 (± 256)	0,003*
Início da TVO				
IGC (semanas)	36 (35 – 36)	34 (33 – 35)	33 (32 – 34)	< 0,01*
Peso (gramas)	1.638	1.735	1.550	0,052
	(1.558 – 1.775)	(1.648 – 1.895)	(1.454 – 1.696)	
Final da TVO				
IGC (semanas)	37 (36 – 38)	35 (35 – 36)	35 (34 – 36)	< 0,01*
Peso (gramas)	1.793	1.841	1.848	0,588
	(1.712 – 1.937)	(1.797 – 1.968)	(1.721 – 2.027)	
		
Tempo total de TVO (dias)	7 (6 – 11)	10 (7 – 18)	12 (11 – 17)	0,009*
				

a Significância da diferença entre os grupos pela Anova ou teste de Kruskal-Wallis

Os dados contínuos estão expressos em média e desvio-padrão ou em mediana e percentis 25 e 75

* Significância em p < 0,05

Abreviações: IG, idade gestacional; TVO, transição para via oral; IGC, idade gestacional corrigida

Houve diferença entre as medianas da idade gestacional corrigida no final da TVO dos grupos estratificados (p < 0,01) (Tabela 1). A análise post hoc mostrou diferenças significativas nas medianas entre os grupos de RNPT tardio e muito pré-termo (35) (p < 0,01) e entre os grupos de RNPT moderado e tardio (p = 0,006), mas não entre os grupos de RNPT moderado e muito pré-termo (p = 0,490).

Com relação ao tempo de TVO foi evidenciada uma diferença entre as medianas dos grupos (p = 0,009) (Tabela 1). A análise post hoc mostrou diferenças significativas nas medianas entre os grupos de RNPT tardio e muito pré-termo (p = 0,007), mas não entre os grupos de RNPT tardio e moderado (p = 0,306) e nem entre os grupos de RNPT moderado e muito pré-termo (p = 0,542).

Dos 52 bebês, 67,3% foram para a UTIN antes de chegarem à UCINCA. Houve diferença entre as medianas do tempo de internação na UTIN dos grupos (p < 0,01) (Tabela 2). A análise post hoc mostrou diferenças significativas nas medianas entre os grupos de RNPT tardio e muito pré-termo (p < 0,01) e entre os grupos de RNPT moderado e muito pré-termo (p = 0,001), mas não entre os grupos de RNPT tardio e moderado (p = 1,000).

**Tabela 2:** Tempo de internação por classificação da prematuridade

**Variáveis**	**Prematuridade**			p-valor^a^
	**Tardio (N=18)**	**Moderado (N=16)**	**Muito pré-termo (N=18)**	
**UTIN (%)**				
Sim	9 (50,0)	8 (50,0)	18 (100,0)	**0,001** *
Não	9 (50,0)	8 (50,0)	0 (0,0)	
**Tempo de Internação (dias)**				
UTIN	1 (0 – 10)	3 (0 – 10)	16 (12 – 25)	p < 0,01*
UCINCA	15 ± 7	16 ± 6	26 ± 9	0,002*
UTIN + UCINCA	20 (15 – 24)	23 (17 – 31)	43 (34 – 48)	p < 0,01*

a Significância da diferença entre os grupos pela Anova ou teste de Kruskal-Wallis (variáveis contínuas) ou teste Qui-quadrado de Pearson (variáveis categóricas)

Os dados contínuos estão expressos em média e desvio-padrão ou em mediana e percentis 25 e 75

* Significância em p < 0,05

Abreviações: UTIN, Unidade de Terapia Intensiva Neonatal; UCINCA, Unidade de Cuidados Intermediários Canguru

O tempo de internação na UCINCA foi estatisticamente diferente entre os grupos de prematuridade (p = 0,002). A análise post hoc de Tukey-Kramer revelou que a diferença do tempo na UCINCA entre os grupos de RNPT muito pré-termo e tardio foi estatisticamente significativa (*p* = 0,002), bem como a diferença entre os grupos dos RNPT moderado e muito pré-termo (*p* = 0,011), porém não houve diferença significativa entre o tempo na UCINCA dos grupos de RNPT tardio e moderado (p=0,914).

Na Tabela 3 estão dispostos os resultados da técnica utilizada durante a transição da alimentação alternativa para a via oral plena e conduta na alta, por classificação da prematuridade.

**Tabela 3:** Técnica utilizada na transição alimentar e conduta alimentar na alta por classificação da prematuridade

Variáveis	Prematuridade			p-valor^a^
	Tardio (N=18)	Moderado (N=16)	Muito pré-termo (N=18)	
**Técnica (%)**				
Translactação	2 (11,1)	3 (18,8)	4 (22,2)	0,667
Sonda-peito	16 (88,9)	13 (81,2)	14 (77,8)	
**Conduta da alta (%)**				
AME	14 (77,8)	12 (75,0)	14 (77,8)	0,976
SM + COMPL	4 (22,2)	4 (25,0)	4 (22,2)	

a Significância da diferença entre os grupos pelo teste Qui-quadrado de Pearson

Abreviações: AME, Aleitamento materno exclusivo; SM, seio materno; COMPL, complemento

## DISCUSSÃO

O peso para se iniciar a TVO ainda é bastante discutido, assim como a idade gestacional corrigida. Esses aspectos são, em algumas unidades neonatais, critérios para se iniciar a oferta da alimentação por via oral dos recém-nascidos prematuros^(8,12,16)^. No presente estudo, observou-se que a mediana da idade de início da TVO da amostra, variou de 32 a 36 semanas de idade gestacional corrigida. Apesar de não ser um critério na maternidade onde a pesquisa foi realizada, a idade gestacional corrigida é um importante indicativo de maturidade da função oral do RN no que se diz respeito a coordenação entre as funções de sucção, deglutição e respiração, fundamental para uma via oral segura^(2,8)^_,_ porém não precisa ser considerada um fator determinante para se iniciar esse processo^(17)^.

Quanto à idade gestacional de nascimento, os resultados do presente estudo sugerem que quanto mais imaturo o recém-nascido, ou seja, quanto menor a idade gestacional ao nascimento, maior será o tempo de TVO. Este fato pode estar relacionado à imaturidade orgânica do RNPT, que traz prejuízo na coordenação entre a sucção, deglutição e respiração e, por isso, a necessidade de vias alternativas de alimentação para se garantir a nutrição^(3)^. Apesar do maior tempo de transição para a alimentação por via oral dos recém-nascidos muito pré-termo, a prevalência de aleitamento materno exclusivo na alta foi semelhante entre os grupos. Este fato demonstra que a orientação de manutenção de extração do leite materno e até mesmo a técnica utilizada pelo fonoaudiólogo para favorecer a alimentação pela via oral, pode ter sido um fator que contribuiu para o favorecimento do aleitamento materno na alta hospitalar, independente da idade gestacional de nascimento do bebê.

Das técnicas utilizadas, observou-se que a técnica sonda-peito foi utilizada com maior frequência. Essa técnica consiste na alimentação da sonda gástrica/gavagem para o peito de forma exclusiva sem uso de copo ou mamadeira^(11)^. Quando esse estímulo acontece de forma segura, ou seja, quando o lactente apresenta padrão adequado de sucção e estabilidade clínica, a TVO ocorre o mais precocemente possível por meio da sucção, reduzindo o tempo de alimentação por sonda e proporcionando a via oral plena^(17)^. Muitas vezes, os RNPT não estão aptos para extraírem a quantidade necessária de leite que precisam para se nutrir. Porém, com o treino de sucção em seio materno, o recém-nascido irá aprimorar a habilidade de coordenação entre a sucção, deglutição e respiração e favorecer a tonicidade e mobilidade dos órgãos fonoarticulatórios^(11,12)^. Na prática clínica a técnica de sonda-peito tem-se mostrado benéfica por proporcionar a sucção sem interferências externas que possam gerar a “confusão de bico” na pega, como o uso de mamadeira e/ou copo, favorecendo o aprendizado contínuo e gradativo da sucção natural em seio materno, tão benéfica para favorecer o adequado desenvolvimento craniofacial da criança, incentivo e manutenção ao aleitamento materno exclusivo, e promover proximidade no binômio mãe-bebê.

A técnica translactação^(17,18)^, utilizada em 17,3% dos RNPT da amostra, tem como objetivos tornar mais fisiológica a adaptação do bebê ao peito, favorecer a produção láctea e estabelecer a coordenação entre sucção, deglutição e respiração. Essa técnica vem sendo utilizada na TVO de RNPT permitindo a associação da ingestão do leite recebido pela sonda com a sucção do bebê na mama^(17,19)^, proporcionando, desta forma, que o aprendizado da função de sucção já aconteça de maneira a favorecer o aleitamento materno nesta população.

Não foi observada oferta de alimentação por copo, mamadeira ou sonda-dedo durante a TVO nesta pesquisa. Por ser um local de Iniciativa Hospital Amigo da Criança (IHAC) existe o incentivo à prática do aleitamento materno por toda a equipe. Visto que a oferta do leite através do copo não é a opção mais fisiológica, principalmente usada a longo prazo, concordamos com que a sucção deve ser estimulada de forma semelhante a sucção em seio materno^(9)^. Para isso, a técnica de translactação, assim como a sonda-peito, são as opções em que o RNPT será treinado e receberá o leite através da sucção direta no seio materno^(19)^.

Na alta hospitalar, a conduta que predominou foi o aleitamento materno exclusivo. Três estudos, com amostras semelhantes à deste estudo, mostram que a prevalência de aleitamento materno de prematuros na alta hospitalar foi de 85,2%^(20)^, 58,3%^(21)^ e 23,3%^(22)^. Os três estudos foram realizados em hospitais com implantação da IHAC, porém, comparando os estudos foi possível observar números desproporcionais de prevalência de bebês em aleitamento materno exclusivo. Vale ressaltar que as estratégias abordadas por toda a equipe de profissionais envolvidos para favorecer o aleitamento materno, assim com uma demanda de pacientes com algum comprometimento físico, com alguma anomalia craniofacial, ou neurológico, são fatores que devem ser levados em consideração na dificuldade de sucesso no aleitamento materno na alta hospitalar. Um destes estudos traz resultado importante, apresentando que mesmo com uma prevalência de 85,7% de RNPT em aleitamento materno exclusivo na alta hospitalar, após 15 dias da alta, a prevalência teve uma diminuição da frequência para 75% e após 30 dias da alta continuou em declínio chegando a 46,3%^(20)^. Reforçando a importância de incluir estratégias para acompanhar o manejo da amamentação mesmo após a alta hospitalar, favorecendo que tanto a mãe quanto o lactente sejam beneficiados com o aleitamento materno exclusivo nos primeiros 6 meses de idade corrigida.

O tempo de permanência em UTIN e UCINCA foi maior no grupo de muito pré-termo. Esse tempo prolongado na UTIN, devido às diversas complicações neonatais consequentes à imaturidade, pode gerar impacto no desenvolvimento neuropsicomotor e é considerado um fator importante para atraso no desenvolvimento por causa da privação sensorial adequada^(23)^. Por isso, o tempo prolongado de internação em UTIN pode ser uma das causas de maior tempo de TVO no grupo de prematuros muito pré-termo. Além de relacionar-se a maiores taxas de aleitamento materno misto^(19)^. Positivamente, na maternidade onde a pesquisa foi realizada, o fonoaudiólogo é o profissional que avalia se a alimentação por via oral do RNPT pode ser iniciada de uma forma segura. O olhar desse profissional na equipe multiprofissional auxilia no processo de TVO para que essa transição seja segura e eficaz, objetivando o aleitamento materno exclusivo na alta hospitalar.

A literatura é escassa no que diz respeito a TVO em prematuros, técnicas utilizadas e prevalência de aleitamento materno nessa população. É importante que haja mais estudos que investiguem as limitações durante esse processo e as dificuldades encontradas para se alcançar o aleitamento materno exclusivo na alta hospitalar.

## CONCLUSÃO

O estudo concluiu que a técnica sonda-peito foi a mais utilizada durante a transição da alimentação da sonda enteral para via oral e que o tempo de transição foi menor para o grupo de recém-nascidos pré-termo tardio e moderado, respondendo positivamente a hipótese de que quanto menor a idade gestacional de nascimento, maior o tempo de transição para via oral.

Também foi possível analisar que a maioria dos recém-nascidos prematuros saiu em aleitamento materno exclusivo na alta hospitalar, respondendo negativamente à hipótese, pois observou-se que as chances de aleitamento materno exclusivo na alta hospitalar foram semelhantes entre os 3 grupos de classificação da prematuridade.
